# Challenges Faced by Rohingya Refugees in the COVID-19 Pandemic

**DOI:** 10.5334/aogh.3052

**Published:** 2020-10-06

**Authors:** Amit Barua, Rutu Hitesh Karia

**Affiliations:** 1 Institute of Applied Health Sciences, Chattogram, BD; 2 Anna Medical College and Research Centre, MU

## Abstract

In August 2017, Bangladesh saw a massive influx of Rohingya refugees following their violent persecution by the Myanmar authorities. Since then, the district of Cox’s Bazar has been home to nearly 900,000 Rohingya refugees living in the densely populated and unhygienic camps. The refugees have been living in makeshift settlements which are cramped into one another, making it extremely difficult to maintain “social distance”. The overcrowded conditions coupled with the low literacy level, lack of basic sanitation facilities, face masks and gloves and limited communication make these camps an ideal place for the virus to spread rapidly. As nations struggle to contain the SARS-CoV-2 virus, refugees are one such population who are extremely vulnerable to the effects of this outbreak. If issues are not addressed at an early stage, its effects can be catastrophic.

In August 2017, Bangladesh saw a massive influx of Rohingya refugees into its territory in Cox’s Bazar following their violent persecution by the Myanmar authorities, which the United Nations described as a textbook example of “ethnic cleansing” [1]. Since then, the district of Cox’s Bazar has been home to nearly 900,000 Rohingya refugees living in extremely harsh, overcrowded and unhygienic conditions. They continue to survive and live under clouds of distress, constantly burdened with the helplessness of being ripped off their identity, the agony of being stateless, the sadness of having no home to call their own and the anguish of losing their loved ones. Three years into their exodus and yet no agreement has been reached on their peaceful repatriation back to their homeland.

However, since early 2020, governments across most nations have imposed lockdowns, shut down businesses, closed schools, and banned all recreational activities and social gatherings, in an attempt to curb the spread of the novel SARS-CoV-2 virus. In addition to these, mandatory measures like use of thermal scanners in workplaces, prohibition of domestic and international travel, and quarantine period for incoming travellers were enforced by the Government of Bangladesh (GoB). Unable to implement these measures successfully due to repeated violations of the lockdown, the government resorted to take help from the armed forces to run quarantine centres, provide emergency services and carry out government provided public health directives within the country. However, a specialised lockdown was levied in Cox’s Bazar which restricted entry into or exit from the district, except for emergency food and medical supplies [2]. Thermal screening and handwashing points at the entry of each camp were placed. The testing capacity for COVID-19 was increased in Cox’s Bazar with the help of the United Nations and other aid agencies. While a vast majority of the world population have confined themselves to the walls of their homes, implementing the new norms of “social distancing” and “handwashing”, one cannot help but ask: how does a refugee incorporate these new norms into their daily life while living in the densely populated camps?

Standing on the top of the hill of Camp-22, all that meets the eye are endless makeshift shelters cramped into one another, with every inch of space utilized to the fullest. Each family, regardless of the size, has a few square meters of land surrounded by walls made of bamboo, to call it their home, their private space. Curtains of silk or plastic sheets divide the interior creating the false illusion of living in separate rooms. Shelters are placed so close to each other that a couple of steps outside the shelter might risk a trespass into the territory of the neighbours. This arrangement makes not only social distancing impossible but also eliminates the possibility of cross-ventilation which is vital to reduce the potential of airborne transmission of COVID-19 [3]. Communal toilets and bathing facilities are installed at various points in the camps. These are to be shared by almost everyone living in the camp. Residents of different tents/households have to share the same facilities as there is one latrine for every 19 persons and one bathing facility for every 41 individuals [4]. While some have soaps and water points installed, others have no provision for any sort of handwashing facilities. To collect water for drinking and daily chores, people gather around water points. To access these facilities, one must walk a few meters, thus making it difficult for women and children to access at night for security reasons. These facilities, being shared by everyone, can serve as a hotspot for the transmission of the virus.

The literacy level of the Rohingya population is very low. Despite being educated by the various aid agencies, they are not able to maintain basic personal hygiene as they have limited access to soaps and water. Handwashing and use of facemasks is not a common practice among this high-risk group of population [5]. Besides, many of them are not aware of the novel virus and its consequences. Humanitarian organizations are facing difficulty spreading information regarding COVID-19 due to the internet ban enforced by the GoB since September 2019, limiting access to mobile data and communications in the refugee camps [6]. This blackout has prevented the dissemination of vital information and delivery of updated knowledge on COVID-19 inside the camps, eventually leading to the spread of misinformation and misinterpretation of facts. Community health workers find it difficult to receive information in the camps regarding any individual suffering from COVID-19 like symptoms such as fever, cough, fatigue, dyspnoea, which form the basic protocol for suspecting and testing an individual for COVID-19 and conveying this information to the concerned authorities. Rumours have been rife among the refugee population regarding COVID-19. Refugees fear being abducted or even killed while being taken to isolation centres [7]. These rumours have prevented many refugees from seeking healthcare despite suffering possibly from COVID-19 symptoms. Turnover in healthcare facilities has dropped; and health authorities are concerned this might lead to the outbreak of other diseases, as patients avoid seeking treatment for their ailments. Rohingya camps are no stranger to outbreaks, having witnessed outbreaks of diphtheria, chickenpox, and measles in the past. In a telephonic survey conducted randomly among the host community and refugee population in Cox’s Bazar, 24.6% of 365 refugees reported having at least one symptom out of fever, dry cough, and fatigue – the three most common symptoms of COVID-19 reported by the World Health Organization (WHO). Among the 120 refugees that sought treatment, 42.3% sought treatment at a pharmacy, followed by 35% by health information providers in camps [8]. Such a fearful and uncertain situation ought to have a debilitating impact on the mental health of an already traumatized and vulnerable population. Hence, WHO, in collaboration with the GoB has had to resort to educating the population on COVID-19 via community health workers, information service centres located in the camps, and announcements through loudspeakers and megaphones, all of which are laborious, time-consuming, and, most important, ineffective.

Besides having limited access to health services, one major challenge faced by refugees is the lack of resources like medicines, face masks, and gloves. With the world economy struggling in the wake of the pandemic, it is feared that humanitarian organizations might face a shortage of funding and supplies. Face masks and gloves, already in short supply throughout the world, might be difficult to make available for the refugees. In a Lancet comment, WHO leaders had appealed for more attention to refugees and migrants, who are facing disruption of essential supplies of food, medicines, and aid workers in this ongoing pandemic [9]. As of 12th July 2020, seven active severe acute respiratory illness isolation treatment centres (SARI ITCs) with 292 SARI and 108 isolation beds are ready to serve both Rohingya refugees and host communities. However, only ten Intensive Care Unit (ICU) and eight High Dependency Unit (HDU) beds are available. A total 981 tests have been conducted in Rohingya refugees, which have led to 57 confirmed COVID-19 cases and five deaths among the refugee population [10]. However, there may be a possibility of a large number of cases being undetected in the camps due to limited testing capacities and lack of social distancing measures in the camp.

Given the above, the vulnerable refugee population continues to be at highest risk for exposure to COVID-19. The present infrastructure of the camps is unsuitable for maintaining “social distance” and the lack of hygiene practice among the population does not help in stopping this virus. Besides, the low level of literacy and lack of awareness among the population makes these camps a potential breeding ground for COVID-19 transmission. All in all, we await the mini-pandemic within the refugee camps, not limited to Cox’s Bazar, but across all parts of the world.
